# Antioxidant Cerium Oxide Nanoparticles in Biology and Medicine

**DOI:** 10.3390/antiox5020015

**Published:** 2016-05-17

**Authors:** Bryant C. Nelson, Monique E. Johnson, Marlon L. Walker, Kathryn R. Riley, Christopher M. Sims

**Affiliations:** 1 Material Measurement Laboratory—Biosystems and Biomaterials Division, National Institute of Standards and Technology, Gaithersburg, MD 20899, USA; christopher.sims@nist.gov; 2 Material Measurement Laboratory—Chemical Sciences Division, National Institute of Standards and Technology, Gaithersburg, MD 20899, USA; monique.johnson@nist.gov (M.E.J.); kathryn.riley@nist.gov (K.R.R.); 3 Material Measurement Laboratory—Materials Measurement Science Division, National Institute of Standards and Technology, Gaithersburg, MD 20899, USA; marlon.walker@nist.gov

**Keywords:** nanoceria, cerium oxide nanoparticles, reactive oxygen species, enzyme mimetics, redox-cycling

## Abstract

Previously, catalytic cerium oxide nanoparticles (CNPs, nanoceria, CeO2-x NPs) have been widely utilized for chemical mechanical planarization in the semiconductor industry and for reducing harmful emissions and improving fuel combustion efficiency in the automobile industry. Researchers are now harnessing the catalytic repertoire of CNPs to develop potential new treatment modalities for both oxidative- and nitrosative-stress induced disorders and diseases. In order to reach the point where our experimental understanding of the antioxidant activity of CNPs can be translated into useful therapeutics in the clinic, it is necessary to evaluate the most current evidence that supports CNP antioxidant activity in biological systems. Accordingly, the aims of this review are three-fold: (1) To describe the putative reaction mechanisms and physicochemical surface properties that enable CNPs to both scavenge reactive oxygen species (ROS) and to act as antioxidant enzyme-like mimetics in solution; (2) To provide an overview, with commentary, regarding the most robust design and synthesis pathways for preparing CNPs with catalytic antioxidant activity; (3) To provide the reader with the most up-to-date *in vitro* and *in vivo* experimental evidence supporting the ROS-scavenging potential of CNPs in biology and medicine.

## 1. Introduction

Cerium (Ce) is a rare earth element (atomic number 58) that belongs to the lanthanide series of the periodic table. Cerium (electron configuration [Xe]4f^1^5d^1^6s^2^) is actually the most abundant of the rare earth metals and is unique in that it can exist in both the +3 (Ce^3+^ = [Xe]4f^1^) and +4 (Ce^4+^ = [Xe]) oxidation states, unlike most of the other rare earth metals which mainly exist in the trivalent state [1,2]. Cerium itself has no biological significance in mammalian physiology, but soluble Ce^3+^ salts (nitrate, acetate, chloride, *etc.*) have been traditionally used by humans for biomedical purposes due to their antiemetic, bacteriostatic, bactericidal, immunomodulating and antitumor activity [3,4]. A commercial dermal burn cream (Flammacerium) [5] formulated from cerium nitrate has recently been granted orphan drug status by the US FDA. The insoluble oxide form of Ce (cerium oxide, CeO_2_, ceria) is not only naturally occurring, but is also manufactured both as a bulk material and as an engineered nanoparticle (NP). Cerium oxide nanoparticles (nanoceria, CNPs), are widely applied in chemical mechanical polishing and planarization processes [6], in the development of corrosion protection coatings for metals and alloys [7] and in diesel fuel oxidation catalysis [8]. Recently, there has been an explosion of fundamental and practical interest in the development and application of CNPs as potential catalytic antioxidants in biology and medicine [9,10,11]. CNPs have generally demonstrated antioxidant enzyme-mimetic activity, as well as having the capacity to actively scavenge a variety of both reactive oxygen species (ROS) and reactive nitrogen species (RNS) in cell and animal models.

The basis for both the enzyme-mimetic and ROS/RNS scavenging capacity of CNPs is dependent, to a certain extent, upon the inherent physicochemical properties of nanoscale materials, the specific ability of CNPs to absorb and release oxygen [12] and the relative thermodynamic efficiency of redox cycling between Ce^3+^ and Ce^4+^ ions on the surface of CNPs [2]. At the bulk scale, cerium oxide can exist as pure CeO_2_ (Ce^4+^) or Ce_2_O_3_ (Ce^3+^). However, at the nanoscale, cerium oxide contains a mixture of both Ce^4+^ and Ce^3+^ on the CNP surface [10]. As the absolute CNP diameter decreases (e.g., from 20 nm to 2 nm), there is a measureable loss of oxygen atoms and an increase in the number of Ce^3+^ sites on the NP surface [6,13,14]. Thus, as oxygen atoms are lost from the CNP surface, there is a reduction in the oxidation state of Ce (Ce^4+^ → Ce^3+^) and an increase in the number of oxygen vacancies (defect sites) on the CNP surface. The ratio of Ce^3+^/Ce^4+^ sites on the surface is strongly correlated with the antioxidant/enzyme-mimetic activity of the CNPs (see next section). The Ce ions are able to switch between the two valence states (Ce^4+^ and Ce^3+^) on the surface of CNPs under aqueous solution conditions. The overall structural stability of the CNPs can best be described using the formula CeO_2-x_ to illustrate the presence of oxygen vacancies under these conditions. Further, each oxygen vacancy must be accompanied by reduction of two surface Ce^4+^ ions to maintain electronic charge stability [6]. Early on, it was postulated that both oxygen vacancy sites and the Ce redox couple (Ce^3+^/Ce^4+^) were equally involved in the CNP biological antioxidant activity [2,7]. However, an elegant experiment performed by Celardo *et al.* utilized samarium (Sm^3+^) doping of CNPs to discriminate between biological antioxidant activity driven by oxygen vacancies *versus* the Ce^3+^/Ce^4+^ redox couple [15]. The experiment was based on the fact that Sm^3+^ is non-catalytic and when Sm^3+^ is substituted for Ce^3+^ in the crystal lattice, the Ce^3+^/Ce^4+^ redox couple is disrupted, but the number of oxygen vacancies is not changed. The researchers found that the Sm^3+^-doped CNPs no longer displayed biological antioxidant activity, providing substantial evidence that the Ce^3+^/Ce^4+^ redox couple is the main location of the intrinsic antioxidant properties of CNPs. The biological significance of the oxygen defect sites in CNPs has not yet been elucidated [6].

In this review, we describe and discuss the putative mechanisms underlying both the antioxidant enzyme-mimetic activity, as well as the ROS-scavenging activity of CNPs. The catalytic antioxidant activities of CNPs are dynamic and sensitive to synthesis procedures, physicochemical properties, chemical environment, *etc.* Hence, a detailed overview of the most common CNP synthesis procedures and resulting physicochemical properties is provided with a description of how these factors relate to antioxidant activity (see Section 3). CNPs have been widely used in various applications in nanomedicine, ranging from the development of novel cancer therapeutics to treatments for Alzheimer’s disease (AD). The most recent accumulated evidence from *in vitro* and *in vivo* studies supporting the use of CNPs as powerful therapeutic antioxidant agents is also presented. Some studies have shown CNPs to have both antioxidant properties, as well as pro-oxidant properties when high CNP doses (≥750 µmol/L) are utilized [16]. CNPs can promote toxic biological responses under certain conditions, but these mechanisms of toxicity, which have been described and discussed previously, will not be discussed in this review. Interested readers are referred to the recent and comprehensive review of Yokel *et al.* [17]. 

## 2. Antioxidant Mechanisms

### 2.1. Antioxidant Enzyme-Mimetic Activity

Oxidative stress is the basis of many serious diseases and one of its primary characteristics is the cellular imbalance between endogenous antioxidant defenses (free radical scavenging by small molecule antioxidants and/or redox enzymes) and ROS (e.g., superoxide radical anion (O_2_^•−^); hydrogen peroxide (H_2_O_2_); hydroxyl radical (^•^OH)) generation inside the cells. CNPs have been shown to be protective in *in vitro* (cells) and *in vivo* (animal) models through the reduction of ROS levels. In the cellular environment, O_2_^•−^ acts as a signaling molecule and is produced as a result of normal cellular metabolism. However, background O_2_^•−^ levels can rise quickly via activation of NADPH oxidases during inflammatory responses and/or disruption of the mitochondrial electron transport chain and interference with ATP production. The toxic effects from excess O_2_^•−^ are normally controlled and reduced through the activity of superoxide dismutase (SOD) enzymes [18] located extracellularly and in the cytoplasm and mitochondria. SOD enzymes eradicate O_2_^•−^ by converting O_2_^•−^ into H_2_O_2_ and O_2_ through a two-step, catalytic dismutation reaction [7]:

(1) O_2_^•−^ + 2H^+^ + (Cu^+^)-SOD → H_2_O_2_ + (Cu^2+^)-SOD
(2) O_2_^•−^ + (Cu^2+^)-SOD → O_2_ + (Cu^+^)-SOD
(3) 2O_2_^•−^ + 2H^+^ → H_2_O_2_ + O_2_ (overall)

The SOD enzyme works by either removing or adding electrons to O_2_^•−^. For example, in step (1) above, the reduced (Cu^+^)-SOD catalyzes the removal of an electron from O_2_^•−^ to generate H_2_O_2_. While in step (2), the oxidized (Cu^2+^)-SOD, (generated in step (1)), catalyzes the addition of an electron to another molecule of O_2_^•−^ to generate O_2_. Reduced (Cu^+^)-SOD is regenerated so that the cycle can begin again. Overall, two O_2_^•−^ molecules are dismutated for every molecule of H_2_O_2_ produced. Similarly, the presumed catalytic mechanism by which CNPs scavenge O_2_^•−^ is reported as follows [6,7]:

(4) O_2_^•−^ + Ce^3+^ + 2H^+^ → H_2_O_2_ + Ce^4+^ (Ce^3+^ is oxidized)
(5) O_2_^•−^ + Ce^4+^ → O_2_ + Ce^3+^ (Ce^4+^ is reduced)

These reactions illustrate the regenerative capacity of the Ce^3+^/Ce^4+^ redox couple, but it is not clear that these are the identical SOD-mimetic reactions that occur with CNPs in actual biological environments. Korsvik *et al.* were the first investigators to demonstrate the SOD-mimetic activity of CNPs [2]. The authors measured the generation of H_2_O_2_ from the catalytic degradation of O_2_^•−^ by CNPs. A key finding from this study was that CNPs prepared with a high Ce^3+^/Ce^4+^ ratio (higher levels of Ce^3+^ on the surface) were more efficient scavengers of O_2_^•−^ than CNPs prepared with lower Ce^3+^/Ce^4+^ ratios on the surface. The importance of having a higher Ce^3+^/Ce^4+^ surface ratio was further established through additional studies which demonstrated that CNPs with low Ce^3+^/Ce^4+^ surface ratios (higher proportion of surface Ce^4+^ ions) were not effective SOD-mimetics [19]. 

High levels of H_2_O_2_ are actually considered more dangerous to cellular homeostasis than high levels of O_2_^•−^ because H_2_O_2_ is involved in the generation of the highly damaging ^•^OH through the Fenton reaction with metals. Catalase, glutathione peroxidases and peroxiredoxins all reduce H_2_O_2_ levels in cells, but catalase (CAT) is the most efficient antioxidant enzyme acting to disproportionate H_2_O_2_ into O_2_ and H_2_O [16]. Rzigalinski and co-workers were one of the first groups to demonstrate the CAT-mimetic activity of CNPs based their studies on observing alterations in the luminescence spectra of CNPs in the presence of H_2_O_2_ added to astrocyte cells [20]. The key findings from this study were that Ce^4+^ was required on the surface of the CNPs in order to decompose the added H_2_O_2_ and that the luminescence spectra of the CNP surface reverted back to its original properties after the H_2_O_2_ was decomposed; this was evidence of the regenerative nature of the Ce^3+^/Ce^4+^ redox couple on the CNP surface. Detailed studies by other investigators have indeed found that CNPs with low Ce^3+^/Ce^4+^ surface ratios [9,21,22] function as efficient antioxidant CAT-mimetics with the following mechanism of action surmised [7,9,21]:

(6) H_2_O_2_ + 2Ce^4+^ + 2OH^−^ → 2H_2_O + O_2_ + 2Ce^3+^

Whether or not a CNP behaves predominantly as a SOD-mimetic or as a CAT-mimetic is critically dependent on the purity of the starting reagents, the synthesis procedure, NP functionalization and a number of other parameters related to CNP preparation and structure (discussed in later sections) [11]. Many CNPs possess both SOD- and CAT-mimetic activity (at varying levels) that are correlated with the presence/absence of oxygen defect sites and Ce^3+^/Ce^4+^ surface ratios. And in general, CNPs with high amounts of Ce^3+^ (40% to 60%) [2,22,23] on the surface perform better as SOD-mimetics while CNPs with high amounts of Ce^4+^ (70% to 80%) [10,21] on the surface perform better as CAT-mimetics.

The potential for CNPs to eliminate noxious levels of O_2_^•−^ and H_2_O_2_ from cellular environments while simultaneously regenerating reduced Ce^3+^ ions on the NP surface (see Equations (5) and (6) above) makes these nanoscale agents ideal SOD- and CAT-mimetics. In fact, CNPs have been considered as possible antioxidant agents for treating disorders and diseases mediated by oxidative stress. However, the exact biochemical mechanisms by which CNPs act as antioxidant enzyme-mimetics inside the cellular environment are still being debated. In addition, the biological significance of the oxygen vacancies in CNPs is still an open question [11]. The lack of consistency in the types of NPs utilized in the various *in vitro* and *in vivo* studies and the generally poor physicochemical characterization of these NPs, as well as the strong influence that different synthesis procedures have on CNP lattice structure and surface reactivity, are all relevant issues that hinder the intrinsic understanding of CNP antioxidant enzyme-mimetic activity [24]. For example, the limited physicochemical characterization data in the literature does not allow us to clearly understand if CNP SOD-mimetic activity is directly/immediately linked to subsequent CAT-mimetic activity in biologically-relevant media (e.g., cell culture media) or even in simple aqueous solutions. To our knowledge, neither CNP reference materials nor reference procedures exist that would allow researchers to properly characterize the physicochemical properties of the NPs used in current studies. Our laboratory is trying to address some of the issues related to the physicochemical characterization (e.g., absolute quantitative determination of surface Ce^3+^ and Ce^4+^ ion levels *versus* CNP size) of CNPs for *in vivo* exposure studies by synthesizing and characterizing CNPs of discrete diameters and shapes using high purity reagents under well-controlled laboratory conditions [25]. We are applying a broad suite of advanced, high-resolution microscopy and spectroscopy tools toward the analytical characterization of these new materials under biologically relevant conditions with the goal of generating characterization data sets allowing comprehensive correlation between the CNP physicochemical properties and the antioxidant and/or biological activity demonstrated *in vivo*. 

The SOD- and CAT-mimetic activities of CNPs have been heavily studied in aqueous environments. Several notable intrinsic and extrinsic parameters critically influence the overall catalytic efficiency of the mimetics. This section reviews the parameters that have been shown to have significant effects on CAT- and SOD-mimetic activity: presence of H_2_O_2_ in solution, presence of phosphate ions ((PO_4_)^3−^) in solution and the solution pH. 

When CNPs are exposed to H_2_O_2_, most if not all of the NP SOD-mimetic activity is terminated [19]. The SOD-mimetic activity does return after all of the H_2_O_2_ is degraded, but regeneration of mimetic activity can take up to 14 d. The postulated mechanism for the H_2_O_2_ effect is based on a series of experiments that showed, via X-ray photoelectron spectroscopy (XPS), that Ce^3+^ on the NP surface was oxidized by H_2_O_2_ to Ce^4+^; this effectively transformed the CNPs such that they had high numbers of Ce^4+^ sites (low Ce^3+^/Ce^4+^ surface ratios), which correlates with weak SOD-mimetic activity.

A number of reports have described how the interaction of (PO_4_)^3−^ anions with CNPs diminishes the SOD-mimetic activity while increasing the observed CAT-mimetic activity [21,26,27]. It is well-established that Ce^3+^ ions form strong coordination complexes with phosphates [1], and it was recently shown that phosphates/phosphorous bind preferentially to CNPs with excess Ce^3+^ sites in comparison to CNPs with excess Ce^4+^ sites [28]. It has been demonstrated that the binding of (PO_4_)^3−^ anions to Ce^3+^ sites generates a CePO_4_-like complex that prevents Ce^3+^/Ce^4+^ redox cycling and essentially stabilizes the CNP in the Ce^3+^ state [26,27]; the ability of CNPs to interconvert between the Ce^3+^ and Ce^4+^ states is critical for maintaining catalytic SOD-mimetic activity. The attention to the effect of (PO_4_)^3−^ anions on CNP SOD-mimetic activity is important because cells and tissues are likely to contain high amounts of phosphate which could have a substantial influence on the CNPs’ biological activity. 

Some controversy exists regarding the effect of pH on CNP antioxidant activity with some studies showing no clear effects [26,27] and other studies demonstrating pronounced effects on both the CNP Ce^3+^/Ce^4+^ redox state and on CNP catalytic antioxidant activity [16,29,30]. When CNPs were incubated in a series of aqueous buffers ranging from pH 3 to pH 9, there was no change in the oxidation state based on ultraviolet/visible (UV/Vis) spectrophotometry nor in SOD-mimetic activity based on the prevention of ferricytochrome reduction by O_2_^•−^ [26]. Xue *et al.* also concluded that pH, over a restricted range from pH 4.7 to pH 7.4, has no obvious role in mediating the antioxidant activity of CNPs [27]. However, these authors did observe a slight change in the amount of Ce^3+^ on the surface of the CNPs as the pH was modified. In contrast, Perez *et al.* showed that the antioxidant activity of CNPs depends directly on the pH of the surrounding medium [30]. Dextran coated-CNPs showed CAT-mimetic activity (degraded H_2_O_2_) in neutral and alkaline solutions, but were inactivated in acidic (pH 4) solutions; the authors also demonstrated this behavior in cellular environments. A more recent study by Karakoti *et al.* appears to confirm these observations through fundamental XPS studies that demonstrate linear changes in Ce^3+^/Ce^4+^ redox state with changes in pH [29]. Some of the discrepancies regarding the pH effects on CNP catalytic activity among the described studies may be due, in part, to the lack of consistency in the type and preparation of CNPs tested in the studies, as well as the propensity for the CNP Ce^3+^/Ce^4+^ ratio to change over time in aqueous oxygenated environments [29]. This provides a good example of why it is prudent to confirm (benchmark) results among different studies using well-characterized reference CNPs with established physicochemical properties if possible. 

In addition to antioxidant SOD- and CAT-mimetic activity, CNPs also exhibit other types of enzyme-mimetic effects *in vitro* and *in vivo*. CNPs have shown phosphatase-like [22,31,32,33,34], oxidase-like [34,35,36,37], peroxidase-like [38] and ATPase-like [22] mimetic activity. As described for the SOD- and CAT-mimetic activity, the Ce^3+^/Ce^4+^ ratio on the surface of the CNPs determines the level of biological enzyme activity.

### 2.2. Antioxidant ROS/RNS Scavenging Activity

CNPs have demonstrated through their antioxidant SOD- and CAT-mimetic activity to effectively reduce O_2_^•−^ and H_2_O_2_ levels, but they have also proven to be efficient scavengers for ROS such as ^•^OH [27,39,40,41] and also for RNS such as nitric oxide radical (^•^NO) [22,42] and peroxynitrite (O_2_NO^−^) [43]. One of the first studies to indirectly suggest that CNPs possess innate ^•^OH scavenging potential was published by Das *et al.* where they showed that CNPs were able to remove ^•^OH formed from H_2_O_2_ in aqueous solutions [39]. Later on, Xue *et al.* provided direct experimental evidence that CNPs efficiently scavenge ^•^OH based on NP size and Ce^3+^ surface levels [40]. These authors performed a simple photometric study with the chromogenic reagent, methyl violet, which showed that as the size of the CNPs decreased and as the level of Ce^3+^ on the surface of the NPs increased (higher Ce^3+^/Ce^4+^ surface ratios), the CNPs became more effective at scavenging ^•^OH and preventing a reduction in the visible absorbance of methyl violet. Another important finding from this study was that the ability of CNPs to reversibly switch from Ce^3+^ to Ce^4+^ is a key factor for their ROS scavenging activity. Based on these observations, the authors postulated the following two step mechanism for the ^•^OH scavenging activity of CNPs, where the first step indicates the oxidation of Ce^3+^ by ^•^OH and the second step indicates the reduction of Ce^4+^ to regenerate Ce^3+^ [40]:

(7) Ce_2_O_3_ + 2[^•^OH] → 2CeO_2_ + H_2_O
(8) 2CeO_2_ (in presence of aqueous H^+^) → Ce_2_O_3_ + ½O_2_

Two recent studies provide further evidence for the ^•^OH scavenging antioxidant potential of CNPs by showing how CNPs can protect DNA from damage induced by ^•^OH attack [27,41]. 

Nitrosative stress is the excessive production of RNS, such as ^•^NO and O_2_NO^−^. Nitric oxide, on its own, is not a particularly reactive molecule [44]. However, when ^•^NO reacts with O_2_ or with O_2_^•−^, it can form a complex variety of highly reactive and damaging species. Specifically, when ^•^NO reacts with O_2_^•−^, it can form O_2_NO^−^, which is a potent oxidizing agent that has high damaging potential for lipids, proteins and DNA [44], similar to the reactivity of ^•^OH. CNPs have been established to be an efficient scavenger of ^•^NO in two recent studies [22,42]. In both studies, CNPs with low Ce^3+^/Ce^4+^ surface ratios were more effective than CNPs with high Ce^3+^/Ce^4+^ surface ratios. The authors postulated the following ^•^NO scavenging mechanism for the CNPs [42]:

(9) Ce^4+^ + ^•^NO → [Ce^4+^ + NO ↔ Ce^3+^ + NO^+^]

CNPs have also been shown to be putative scavengers (decomposers) of O_2_NO^−^ or at least of O_2_NO^−^ breakdown products, such as the carbonate radical anion (CO_3_^•−^) [43]. Interestingly, the scavenging proficiency of CNPs for O_2_NO^−^ did not correlate with either low or high Ce^3+^/Ce^4+^ surface ratios; both low and high Ce^3+^/Ce^4+^ ratios accelerated the decomposition of O_2_NO^−^. The authors of this study could not postulate a scavenging mechanism due to the complex biochemistry of O_2_NO^−^.

## 3. Design and Synthesis of ROS Scavenging CNPs

CNPs have been prepared by a number of synthetic processes, including but not limited to, aqueous precipitation [22], solvothermal [45], microemulsion [46] and sol-gel [47] methods. Each of these methods utilize different parameters that control the rates of nucleation and growth, the two principal steps in the NP crystallization process that determine the outcome of a particular NP synthesis procedure [48]. These parameters include the selection and concentrations of precursors, stabilizing agents, solvent, the duration of reaction, the reaction temperature, *etc.* While the sheer number of these variables can complicate the synthesis process, shrewd utilization allows a considerable amount of control over the desired result. This enables the ability to selectively produce CNPs with specifically targeted physicochemical properties (e.g., size, shape, surface coating, surface charge, oxidation state, *etc.*).

A variety of methods have been reported for preparing CNPs with specific physicochemical properties. Many begin with the use of a cerium (III) salt precursor (most commonly cerium nitrate or cerium acetate), which is then oxidized in some fashion to form ceria and hence, CNPs. Zhang *et al.* devised a simple precipitation method where CNPs with sizes between 3 nm and 12 nm and having narrow size distributions could be selected by simply controlling the reaction time [49,50]. Merrifield and Wang *et al.* demonstrated similar size selectivity by modifying the chain length of the polyvinylpyrrolidone (PVP) coating ligand; using longer PVP chain lengths resulted in larger CNPs [51]. Several reports have demonstrated synthetic control of CNP geometry, with spheres [22], octahedrons [50], cubes [52], rods [53] and wires [54] amongst the different morphologies prepared. Each of the different CNP morphologies have varying concentrations of crystal facets (e.g., (111), (110), (100)) and each of these different facets have different stabilities/reactivities [55]. CNPs have been functionalized with a variety of coatings, which can also be used to control the CNP size and shape during the synthesis process [24]. Examples of common coatings include: poly(acrylic acid) (PAA) [56], polyethylene glycol (PEG) [57], dextran [30], and polyethylenimine (PEI) [58]. In addition, the surface charges of CNPs can vary by way of the coating material, an example of which are the negatively-charged PAA-coated CNPs synthesized by Asati *et al.* that could be modified to have positively-charged amine-termini [59]. The Seal research group has demonstrated the ability to control the Ce^3+^/Ce^4+^ oxidation state ratios of synthesized CNPs in multiple reports [2,19,21,60].

Synthetic control over the physicochemical properties of CNPs, such as those described previously, is essential because these properties directly influence the interactions of CNPs with themselves and with other entities. For antioxidant applications, this is critical, as differences in even one of these properties can significantly alter the material, potentially resulting in undesirable effects. For example, a study by Deshpande *et al.* found that the Ce^3+^/Ce^4+^ oxidation state ratio is dependent on the primary particle size, with smaller CNPs having increased concentrations of Ce^3+^ compared to their larger counterparts [12]. Zhang *et al.* showed that the (100) crystal facet was more reactive than the (110) facet by investigating the shape effects of CNPs, with both planes having increased antioxidative activity over the most thermodynamically stable (111) facet [41]. Asati *et al.* demonstrated how CNPs could be transformed from having antioxidant properties to being pro-oxidants by simply lowering the pH of the CNP media [36]. McCormack *et al.* reported that dextran-coated CNPs were more effective in reducing the loss of CNP redox capacity than their PEG-coated counterparts when in the presence of phosphate ions [57,61]. Our progressive understanding of how the physicochemical properties of CNPs impact their antioxidant activity is illustrated in the following examples, where CNPs with specifically designed physicochemical properties are produced to yield beneficial, antioxidative effects.

The research teams of Seal and Self have published several reports on the use of CNPs as antioxidants [42,62,63]. Of particular note is the study by Pirohamed *et al.* where the CAT-mimetic activity of CNPs was shown to be dependent on their Ce^3+^ and Ce^4+^ oxidation state ratios [21]. Here, two similar, yet distinct synthesis methods were used to produce CNPs with different Ce^3+^/Ce^4+^ ratios. While both reactions used cerium nitrate as a precursor, CNPs with increased Ce^3+^/Ce^4+^ ratios (higher Ce^3+^ content) were prepared using H_2_O_2_ as the oxidizing agent, while the CNPs with lower Ce^3+^/Ce^4+^ ratios (higher Ce^4+^ content) were prepared using ammonium hydroxide as the initiator for CNP formation [2,62]. In their experiments, the CNPs with increased levels of surface Ce^4+^ atoms were found to more rapidly convert H_2_O_2_ into O_2_. In contrast, the CNPs with higher Ce^3+^ concentrations were less efficient at this process. Interestingly, the results of this work on CAT-mimetic activity contrast with their previous study of CNPs for SOD-mimetic activity, where the opposite correlation was found [2]. That is, the SOD-mimetic activity of the CNPs increased with increased concentrations of Ce^3+^ and decreased with increased amounts of Ce^4+^. However, the two studies are quite complementary; combined, these studies illustrate how the oxidation states of CNPs can dramatically influence their ability to function as antioxidants and also provided insight into the potential mechanisms of their antioxidative activity [9].

An elegant procedure was developed by Lee *et al.*, where water-stable CNPs with various coatings could be prepared from a single, nonpolar-phase synthesis [58]. Here, the initial oleylamine coating of the CNPs could be exchanged with various water-stable ligands with the assistance of phase transfer agents. In their study, the authors used a modified version of their previous method [64], to prepare CNPs of three different sizes (3.8 nm, 5.4 nm, and 8.2 nm), with each having four different coatings to investigate the impact of these variables on the CNPs’ antioxidant properties. Using a colorimetric assay, they determined that the smaller the CNPs, the larger the absorption change after addition of H_2_O_2_, correlating with the greatest antioxidant capacity; these findings were consistent regardless of the identity of the CNP coating. The increased concentration of Ce^3+^ in the smaller CNPs (as previously described) was given as the reason behind the increased antioxidant activity. Furthermore, Lee *et al.* [64] found that the CNP coating also had an effect on the CNPs’ antioxidant activity. Here, it was discovered that the thickness of the particle coating strongly affected how much H_2_O_2_ could reach the CNP surface, with CNPs coated with thinner surface stabilizers quenching more H_2_O_2_ than their counterparts coated with thicker stabilizing agents. *In vitro* studies of the smallest CNPs coated with the thinnest surface stabilizer were also performed in fibroblasts, where these particular CNPs were shown to be potent antioxidants with low toxicity. Although the CNPs were functionalized by both positively- and negatively-charged coatings, the influence of particle surface charge on H_2_O_2_ quenching was not investigated by the authors. Given previous demonstrations of surface charge effects [59,65], further study of the influence of surface charge on antioxidant activity would be interesting and informative.

The very recent study by Kwon and Cha *et al.* described the preparation of CNPs capable of localization into the mitochondria of various cell lines [66]. Once localized, these CNPs were not only able to efficiently scavenge mitochondrial ROS *in vitro*, but were also able to reduce oxidative stress in an *in vivo* mouse model (as will be discussed in later sections). In order to achieve these useful abilities, a two-step sol-gel method was used to first prepare the CNPs, and then functionalize them with a polymer terminated with positively-charged triphenylphosphonium ion (TPP) groups to achieve water-dispersibility while maintaining hydrophobicity. Through a series of step-wise experiments, it was determined that a number of physicochemical factors were essential for the CNPs’ ability to enter the cell mitochondria. Specifically, the CNPs needed to (1) have small primary particle sizes *and* small hydrodynamic diameters (to be small enough to cross the mitochondrial membrane); (2) maintain good colloidal stability (to prevent effective size-increasing agglomeration); and (3) possess a positive ζ–potential and hydrophobicity (to target and cross the negatively-charged mitochondrial membrane). Once localized in the mitochondria, the small sizes of the CNPs allowed them to provide their antioxidative effects, as demonstrated in the aforementioned studies.

Despite the advances in CNP designs for antioxidant activity, their complex chemistry has resulted in the observation of both antioxidative and pro-oxidative effects. For example, despite the findings of the above reports, where the beneficial antioxidative effect was attributed to increased Ce^3+^ concentration, other studies have suggested that an increase in Ce^3+^ concentration results in increased harmful effects [67]. The existence of discrepancies such as these suggests that the understanding of the physicochemical properties of CNPs and their influence on antioxidative activity has not yet reached a consensus and warrants further research. Robust analytical characterization of newly synthesized CNPs with corresponding biological activity comparisons against well-characterized CNP reference materials would be helpful in achieving this goal [25].

## 4. CNP Antioxidant Activity—Evidence from *in Vitro* Studies

Ongoing research efforts have produced findings that bolster the role of CNPs as an efficient antioxidant in *in vitro* studies. In recent years, CNPs have been shown to have ROS scavenging capabilities that provide protection in several cell models including gastrointestinal epithelium [68], human breast line [69], neuronal [70,71], endothelial [72] and stem [73].

Amyloid beta plaques have been found to be a possible cause of AD through abnormal production of ROS [74,75]. Recently, Kwon and Cha *et al.* [66] elucidated the therapeutic candidacy of CNPs to mitigate oxidative stress in AD. In their study, conjugated CNPs were mainly localized to mitochondria, suppressing neuronal death (which correlates to the severity of memory deficits), and mitigating reactive gliosis and mitochondrial damage in an AD model. CNPs have been found to be of great interest as a therapeutic treatment for ocular diseases as it was shown to protect retinal function by decreasing ROS, but also play an integral role in up-regulating the expression of neuroprotection-associated genes, and down-regulating apoptosis signaling pathways [76]. Embedding CNPs into poly(lactic-co-glycolic acid) films improved the attachment and spatial growth of adult cardiac stem cells, demonstrating a benefit of CNPs within the biografting field [73]. Heparin-functionalized CNPs showed an improvement in ROS scavenging ability as compared to uncoated CNPs and resulted in an enhancement of uptake, an increase in proliferation, and a reduction in intracellular ROS in activated human monocyte cells, U937 [77]. 

Sack *et al.* [78] demonstrated that CNPs enhanced the antitumor activity of the chemotherapeutic agent doxorubicin in human melanoma cells. Cytotoxic effects were exhibited in human melanoma cell line A375 after co-incubation with doxorubicin and CNPs, with a resulting decrease in cell viability of melanoma cells. Additional synergistic effects were seen as the CNPs mitigated the cytotoxicity of doxorubicin in normal cells. CNPs showed antitumor activity, inducing apoptosis in A375 cells, but did not induce DNA damage, a side effect seen with other treatments that often increases the risk of secondary cancer. This study demonstrated the potential for CNPs to serve as an adjuvant therapy to chemotherapy, as CNPs may lower the deleterious side effects on healthy cells during traditional chemotherapy. The authors rely heavily on the synergistic effect of CNPs combined with doxorubicin, however they did find that interaction with CNPs alone caused cytotoxic effects with a decrease in cell viability by approximately 88% at 300 µmol·L^−1^ after 48 h incubation when compared with untreated control samples. In addition, CNP treatment caused an increase in ROS formation in A375 cells, which was mitigated by the co-incubation of CNPs and doxorubicin. Given these results, careful pre-incubation of doxorubicin and CNPs must be performed in order to mitigate the cytotoxic effects before this form of treatment can be considered viable for potential cancer therapeutics. 

Ciofani *et al.* [79] examined and reported the effects of incubation of PC12 neuron-like cells with increasing concentrations of CNPs. PC12 cells showed no deficiencies in viability or metabolic activity, but retained cell differentiation capabilities. Additionally, exposure to CNPs led to an increase in neuronal length, reduced the production of ROS in cells stimulated with H_2_O_2_, and increased the production of dopamine in a dose-dependent manner. Akhtar *et al.* [80] utilized human breast cancer (MCF-7) and fibrosarcoma (HT-1080) cells to explore the potential cytotoxicity of CNPs. They observed no significant cell death in either cell model following treatment with 20 µg·mL^−1^, 50 µg·mL^−1^, 100 µg·mL^−1^ and 200 µg·mL^−1^ concentrations of CNPs and cell viability was maintained under these conditions. CNP-treated cells significantly increased the production of glutathione (GSH), integral in cellular defense against injury, and remarkably replenished GSH depletion caused by H_2_O_2_.

Di Nardo and Traversa [70] evaluated the ability of CNPs (5 nm to 8 nm) to aid in controlling oxidative stress and proliferation of adult cardiac progenitor cells. An attenuation of oxidative effects was exhibited for cardiac progenitor cells pre-treated with 50 µg·mL^−1^ CNPs and exposed to H_2_O_2_ for 24 h. Remarkably, a reduction in ROS production was exhibited at all CNP concentrations (10 µg·mL^−1^, 25 µg·mL^−1^ and 50 µg·mL^−1^) when H_2_O_2_ was added to the cell culture medium 7 days after CNP pre-treatment. Internalized CNPs, in the form of aggregates and localized in the cytosol 7 days after incubation with cells, did not interfere with cardiac progenitor cell morphology, induce functional cell modifications, cause cell structural damage or affect survival or growth *in vitro*. A time-dependent decrease in ROS levels was found at the highest treatment concentration over 7 days, while smaller CNPs required longer activation times to generate the antioxidant function. This data suggests that CNP treatment could be incorporated into progenitor cell reconstruction protocols to aid in the maintenance of ROS balance and assist in DNA repair as the CNPs showed minimal harmful effects to the cells. The authors demonstrated the long-term antioxidant action of CNPs *in vitro* and the capability of regenerative antioxidant activity after a single CNP dosage, which could essentially halt intracellular ROS generation. Further work is needed to ascertain optimal culturing conditions, antioxidant activation time, dosing concentrations, the long-term effects of CNP incorporation into cells and CNP clearance.

Marcos *et al.* [81] investigated the antioxidant and anti-genotoxic effects of CNPs using a human epithelial lung cell line (BEAS-2B) as a model system. They found that internalization of CNPs into BEAS-2B epithelial lung cells did not induce significant cell mortality, and CNPs exhibited protective effects after exposure of pulmonary cells to the well-known oxidizing agent, KBrO_3_. Pre-treatment of cells (24 h) with CNPs reduced mortality, diminished intracellular ROS generation to levels close to that of untreated cells and increased the expression of antioxidant genes such as *Ho1*, *Sod2*, and *Gstp1*. The increase in the expression levels of the antioxidant genes confirmed that the CNPs were protective and indirectly allowed the cells to rebound in response to ROS insult [82]. Additionally, BEAS-2B cells exposed to CNPs at concentrations ranging from 10 µg·mL^−1^ to 90 µg·mL^−1^ were >90% viable 24 h and 48 h post-exposure when compared to untreated cells. This study successfully demonstrated the connection between the ability of CNPs to scavenge cytoplasmic ROS and the inhibition of oxidatively-induced DNA damage in the nucleus. The results from this study illustrate the potential benefits of utilizing CNPs as effective agents against diseases linked to oxidative stress.

Despite the deleterious effects that have been reported [83,84,85], CNPs targeted for therapeutic purposes are distinguished from other engineered nanomaterials used in cancer therapy by their undeniable antitumor capabilities. CNPs promote oxidative stress in malignant cells, suppress cancer cell proliferation and induce apoptosis, while, most importantly, protecting healthy normal cells from damage induced by oxidative stress [86].

## 5. CNP Antioxidant Activity—Evidence from *in Vivo* Studies

Expanding on the promising results from *in vitro* studies, CNPs have been evaluated *in vivo* for their efficacy as treatments in a variety of disease states, with potential medicinal applications ranging from reproductive [87] to gastrointestinal [88] to ophthalmologic [76,89,90,91] and to neurological [66,92] health. Animal and plant models have been used to assess both the potential therapeutic benefits and the potential toxicity of CNPs either intentionally administered or unintentionally present in the environment. Although toxicity assessments of CNPs are not the focus of this review, relevant information regarding uptake and biodistribution of CNPs gleaned from these studies will be included where appropriate.

### 5.1. Uptake and Biodistribution of CNPs in Vivo

*In vivo* animal studies using rats and mice overwhelmingly revealed uptake of CNPs primarily in the liver and spleen, to some degree in the kidneys and lungs, and to a much smaller degree in the brain [93,94,95,96,97,98,99]. In some cases, agglomeration of CNPs was observed [94,98], although it has yet to be determined whether the CNPs were taken up as agglomerates or whether agglomeration occurred within the organ following uptake and to what extent agglomeration influenced uptake. Yokel *et al.* reported time- and dose-dependent uptake of ~30 nm CNPs in the liver and spleen [94] and expanded upon this in a later study to include the effects of size (5 nm *vs.* 30 nm), shape (cubic/polyhedral *vs.* rod), and dose frequency and concentration. In this latter study, no significant differences were observed with regard to biodistribution and retention [96]. 

Most studies report low or undetected levels of CNPs in the brain [93,94,96,97,98], although certain formulations of CNPs, with added stabilizers, may allow entry through the blood brain barrier (BBB) [92]. Specifically, CNPs stabilized with citrate and ethylenediaminetetraacetic acid (EDTA) exhibited differences in biodistribution, including potential crossing of the BBB, relative to unfunctionalized CNPs. It is presumed that these differences resulted from reduced protein adsorption on the CNP surface yielding a unique protein corona for citrate/EDTA-stabilized CNPs compared to unfunctionalized CNPs. Yet another study, which utilized *in situ* brain perfusion to deliver CNPs to rats, showed that when cerebral capillaries were isolated from brain parenchyma, the majority of CNPs were associated with the surface of capillary endothelial cells and it is unknown at that stage whether uptake by cells occurs or whether the CNPs are redistributed back into circulating blood [95]. The success of CNP delivery to the brain parenchyma is believed to be due to the brain perfusion technique, which does not allow the CNPs to mix with blood (at least upon introduction), where otherwise they would be susceptible to the formation of a protein corona. Each of these studies demonstrates the role that proteins associated with the particle surface can play in the distribution of CNPs, and also provide some unique ways to circumvent issues regarding the formation of protein coronas.

Significantly fewer studies have evaluated the uptake of CNPs in non-rodent models. In one study, *Caenorhabditis elegans* (*C. elegans*) were exposed in cell culture medium to CNPs with differently charged surface coatings. In all cases, particle uptake was observed, but positively charged particles had significantly higher bioaccumulation than neutral or negatively charged particles [100]. The authors also showed that the bioaccumulation of CNPs decreased in the presence of excess humic acid, complicating the assessment of uptake in organisms found in complex environments [100]. *Drosophila melanogaster* (*D. melanogaster*) were also examined as model organisms for evaluating CNPs *in vivo*, and uptake of CNPs was demonstrated in the interior of the intestine, in the microvilli and cytoplasm of intestinal cells, and in the intestinal lumen and hemolymph tissues, suggesting that CNPs ingested as food are able to pass through the intestine and be absorbed by midgut cells [101].

Several *in vivo* plant studies have demonstrated the uptake and biodistribution of CNPs in a number of crops, including wheat [102], pumpkin [102], sunflower [102], kidney bean [103], cucumber [104,105], radish [106,107], tomato [105], corn [105], alfalfa [105] and rice [108]. Generally, CNPs were shown to have higher uptake in plant roots relative to other parts of the plant (e.g., leaves, shoots), with some translocation of CNPs from roots to shoots reported [102,103,106]. CNP uptake and distribution is believed to be affected by factors such as CNP size [102,104,106], concentration [103,104,105], agglomeration [104] and transformation (e.g., to yield insoluble Ce compounds like CePO_4_ [102]). The majority of these studies sought to assess the potential environmental impact of CNPs, so the reader is directed to the primary literature for further discussion.

### 5.2. Regenerative and Therapeutic Potential of CNPs

As mentioned previously, the regenerative redox activity of CNPs potentiates their use as treatments for diseases associated with the production of ROS. For example, *D. melanogaster* larvae exposed to cell culture media doped with CNPs exhibited an inverse dose-dependent relationship between the concentration of CNPs administered and the ROS level in hemocytes, demonstrating the radical scavenging potential of CNPs [101]. It has been further demonstrated that CNPs may undergo *in vivo* processing in organs like the liver, and that the associated transformation of the CNPs may have long-term protective effects due to their increased ROS scavenging ability post-transformation [109].

Expanding on these principles, researchers have shown that CNPs may have potential anti-inflammatory [62,110] and wound healing [111] properties. Inhibition of lipid peroxidation (LPO) and a decrease in oxidative stress markers were observed in CNP-treated mice and the effects were comparable to mice treated with the widely used antioxidant, *N*-acetyl cysteine (NAC) [110]. CNPs applied locally to incised rats enhanced wound healing activity with regard to hydroxyproline content, wound tensile strength, and wound closure time relative to treatment with the well-known antiseptic, povidone-iodine [111]. These enhancements were attributed to the ROS-scavenging abilities of the CNPs at the site of injury and protection of the native tissue by increased production of collagen and hydroxyproline. 

Several studies report the regenerative effects of CNPs for a range of diseases. Treatment of mice with induced endometriosis resulted in decreases in ROS levels, angiogenesis, density of endometriosis glands and microvessels and in lesions that negatively impact pregnancy [87]. Similarly, mice injected with human malignant melanoma cells and treated with CNPs had ≥70% reduction in tumor volume and weight compared to control studies [112]. Further work has shown that treatment with CNPs can protect gastrointestinal mucosa from oxidative damage in rats with ethanol-induced gastric ulcers [88]. One preliminary study even suggests that the antioxidant properties of CNPs could be used to combat obesity, noting an initial reduction in the body weight of rats over the first 3 weeks of the study and with an overall increase in weight of just 2% in total body weight over the 6 weeks of the study compared to a 13% increase in the weight of control rats over the same time period [113]. Other noteworthy applications include the use of CNPs to treat photoreceptor degeneration [76,89,90,91] and neurodegenerative diseases, like multiple sclerosis (MS) [92] or AD [66], and these will be discussed in greater detail below.

### 5.3. Treatment of Photoreceptor Degeneration Using CNPs

McGinnis and coworkers have conducted several studies to determine whether CNPs could be used to reduce photoreceptor degeneration with potential implications for the treatment of a number of blinding diseases including age-related macular degeneration, diabetic retinopathy, and retinitis pigmentosa [76,89,90,91]. Early studies employed *tubby* mice [76,90], which have a genetic mutation that causes inherited retinal and cochlear degenerations. The antioxidant properties of CNPs (injected intracardially) showed remarkable regenerative effects including improved neuronal response to light, slowing of photoreceptor degeneration and inhibition of caspase-induced apoptosis. ROS levels in CNP-treated mice were reduced almost to control levels and antioxidant-associated proteins were upregulated along with proteins associated with pathways that protect photoreceptors [76]. Later, it was shown that retinal function was preserved for up to 6 weeks following intravitreal administration of CNPs, over which time untreated *tubby* mice would have typically lost about two thirds of photoreceptor cells in the retina. Interestingly, it was also shown that rod function was retained longer than cone function (~95% to 97% of photoreceptor cells are rods) [90].

Building on this work, it was later shown that ~90% of CNPs remained in the eye up to 120 days following injection of rats with CNPs, although the applied dose was much higher (1000×) than the effective dose needed, since it was unknown how quickly CNPs would be cleared in the eye [91]. Distribution of CNPs was observed primarily in the retina, but also in the lens and eyecup 1 h post-injection [91]. These studies were subsequently extended to another animal model, P2H31 rats, which are a model for retinitis pigmentosa. Similarly, delayed rod cell degeneration, decreases in apoptosis of photoreceptor cells, and decreases in lipid peroxidation in the retina were observed for CNP-treated rats [89].

This body of work has demonstrated the regenerative antioxidant activity of CNPs, including reduction in apoptosis of photoreceptor cells, reduction in oxidative stress and prolonging of rod and cone function *in vivo*. The radical scavenging activity of CNPs is believed to be the primary mechanism in lowering oxidative stress, which prolongs the life of post-mitotic rod photoreceptor cells. Approximation of CNP half-lives suggests that a single dose of CNPs could have prolonged effects over several months or even a year. Taken together, these studies show significant potential for the use of CNPs as ophthalmic treatments.

### 5.4. CNPs as Therapies for Neurodegenerative Diseases

Many of the studies describing the uptake and biodistribution of CNPs *in vivo* suggest that the brain does not easily take up CNPs. Still, some researchers have explored the use of CNPs for the treatment of neurodegenerative diseases. One study involved the subcutaneous injection of mice with chronic progressive experimental autoimmune encephalomyelitis (EAE), a murine model for MS [92]. The efficacy of citrate/EDTA-stabilized CNPs was compared to fingolimod, which is a current treatment for MS. CNPs were delivered either as a preventative treatment (before injection of EAE) or as a therapeutic treatment (following injection of EAE), and in all cases (including fingolimod treatments) maintenance dosing was administered. Uptake of CNPs was observed in the cerebellum (typically a region with significant damage in EAE animals) and further analysis of the cerebellum revealed CNPs distributed throughout the intracellular compartments (e.g., axons, mitochondria), with additional evidence suggesting that the CNPs may cross the BBB. Resultantly, CNP treatments delayed disease onset, and in particular, the pre-treatment dose of CNPs demonstrated comparable efficacy to fingolimod at the same concentration, although fingolimod was capable of delaying disease onset even when administered after induction of EAE. Further improvements were observed in the motor functions of the mice and a reduction in ROS levels of ~ 31% compared to control and fingolimod-treated mice.

Kwon and Cha *et al.* explored the use of TPP-CNPs, designed to target the mitochondria, as a potential therapy for AD [66]. It is believed that AD pathogenesis is due, at least in part, to ROS-induced impairment of mitochondria. As previously mentioned (see Section 3), the small hydrodynamic diameter, colloidal stability, positive zeta potential and hydrophobicity of TPP-CNPs were all reported to play a role in the localization of the NPs in the mitochondria. The subiculum of a transgenic AD mouse model was injected stereotactically with TPP-CNPs and neuronal loss and Aβ plaque accumulation (two markers of AD) were monitored in treated and untreated mice. Two months after treatment, neuronal loss was significantly restored in TPP-CNP-treated mice, however no differences were observed in the accumulation of Aβ plaque in the subiculum, suggesting that neuronal restoration is independent of Aβ plaque accumulation. Abnormal glial activation was also studied to better understand how TPP-CNPs moderate neuronal cell death and results suggest that reduction of mitochondrial ROS may be the mechanism by which TPP-CNPs prevent brain inflammation.

Overall, there is considerable evidence for the uptake of CNPs *in vivo* and their role as antioxidant and/or anti-inflammatory agents to mitigate a variety of diseases. Some studies suggest that the modification of these particles with different stabilizers or surface functional groups may enable targeted delivery of CNPs in the body. Further, many reports demonstrate the long half-life of CNPs, thereby minimizing the necessary dosing requirement. Collectively, CNPs have significant clinical potential as regenerative treatments for diseases particularly characterized by oxidative stress and inflammation.

## 6. Future Perspectives

From the studies and applications described in the present review, it is clear that the development and use of antioxidant CNPs in both biology and medicinal research is expanding at a rapid pace. CNPs are already being tested in both *in vitro* and *in vivo* models as a potential treatment modality for a variety of oxidative-stress/nitrosative-stress related conditions for various types of cancers [68,69,114,115,116,117], ocular diseases (macular degeneration, retinoblastoma, glaucoma, *etc.*) [76,90,91,118,119,120,121], neurodegenerative diseases [39,92,122,123], chronic inflammation [62,124,125], ischemic cardiomyopathy [126], endometriosis [127] and diabetes [128]. At the moment, CNPs occupy a unique niche in relation to their singular ability to act as regenerative antioxidants in the treatment of cancers and ocular diseases. 

For example, when ionizing radiation is used as a therapy to treat cancer, the radiation produces high levels of intracellular and extracellular ROS, including O_2_^•−^ and H_2_O_2_. Accordingly, recent studies have demonstrated that CNPs protect normal cells, but not cancer cells from radiation induced damage [68,116,117]. One probable rationale for this behaviour is based on the significant effect that pH has on the antioxidant enzyme-mimetic activity of CNPs (as discussed in Section 2) [30]. When CNPs are taken up by lysosomes in cancer cells, they encounter an acidic pH microenvironment (pH 4 to pH 5) [59] that inhibits their CAT-mimetic (H_2_O_2_ decomposition) activity, but not their SOD-mimetic (O_2_^•−^ dismutation) activity [115]. The H_2_O_2_ levels thus build up in the cancer cells after the ionizing radiation, leading to the increased generation of the highly damaging ^•^OH; the cells then die via the apoptotic pathway. In normal cells on the other hand, CNPs are not taken up by lysosomes [59] and usually only encounter the neutral pH (pH 7.4) environment present in the cytoplasm, which does not significantly alter their ability to decompose both O_2_^•−^ and H_2_O_2_ through their inherent SOD- and CAT-mimetic activities. Based on the demonstration of site-selective activity, these studies point to the potential future use of CNPs in personalized nanomedicine applications such as precision oncology treatments exclusively tailored to the individual. 

Regarding the treatment of ocular diseases, CNPs have proven to be extremely effective in trapping ROS in several different animal models (previously discussed) and preventing or slowing down the progression toward blindness. Selective degeneration of retinal neurons and/or pathological neovascularization from ROS activity in the eye is a major cause of blindness [10]. In a recent study, CNPs (344 ng) were intravitreally injected into the eye of an adult rat and found to localize in retina cells with a retention half-life well over 1 y (414 d) [91]. In addition, injection of the CNPs into the eye did not lead to any detectable acute or chronic adverse effects. The positive outcomes from this uptake study, in combination with the positive findings from other ocular studies that illustrate efficient ROS scavenging in retina photoreceptors, strongly suggest that CNPs may be able to provide long lasting protection against a multiplicity of ocular diseases.

Despite these advances, much is still to be learned about the interactions between CNPs and biological entities, one of which is protein association (*i.e.*, protein corona formation). As previously mentioned, NP exposure to biological fluids can result in the formation of an encompassing protein corona that can influence their biodistribution [92,95,129]. In addition, the nature of the protein corona can also vary, with proteins with a higher affinity for the NP surface forming an irreversibly bound “hard” corona, while proteins with lower affinities form a more loosely associated “soft” corona [130,131]. Interestingly, the presence of the corona may not affect the antioxidant properties of CNPs, as shown where CNPs immersed in the cytoplasmic matrix still demonstrated cytoprotective antioxidant activity [132]. The exact biological mechanisms and CNP physicochemical characteristics behind these observations (e.g., selective uptake into cancer cells and not normal cells, protein corona formation, *etc.*) are still not fully known and warrant further research.

## 7. Conclusions

In summary, to ensure that the ROS-scavenging CNPs fulfill their promise as alternative or co-therapies for oxidative-stress and nitrosative-stress related diseases and disorders, it is essential that well-characterized CNP reference materials be used in future disease model studies to help clarify the true *in vivo* antioxidant mechanisms. The necessary reference materials do not currently exist, but their development and application may help clarify any biological effects, beneficial or adverse, that may result from the long-term use of CNPs in biology and medicine.

## Abbreviations

The following abbreviations are used in this manuscript:

ATP	Adenosine Triphosphate
AD	Alzheimer’s Disease
BBB	Blood Brain Barrier
CAT	Catalase
CNPs	Cerium Oxide Nanoparticles
DNA	Deoxyribonucleic acid
EDTA	Ethylenediaminetetraacetic Acid
EAE	Experimental Autoimmune Encephalomyelitis
GSH	Glutathione
LPO	Lipid Peroxidation
MS	Multiple Sclerosis
NAC	*N*-acetyl Cysteine
NPs	Nanoparticle(s)
NADPH	Nicotinamide Adenine Dinucleotide Phosphate
PAA	Poly(acrylic acid)
PEG	Polyethylene Glycol
PEI	Polyethylenimine
PVP	Polyvinylpyrrolidone
RNS	Reactive Nitrogen Species
ROS	Reactive Oxygen Species
SOD	Superoxide Dismutase
TPP	Triphenylphosphonium ion
XPS	X-ray Photoelectron Spectroscopy
